# Automatic detection of diffusion modes within biological membranes using back-propagation neural network

**DOI:** 10.1186/s12859-016-1064-z

**Published:** 2016-05-04

**Authors:** Patrice Dosset, Patrice Rassam, Laurent Fernandez, Cedric Espenel, Eric Rubinstein, Emmanuel Margeat, Pierre-Emmanuel Milhiet

**Affiliations:** Inserm, U1054, Montpellier, France; Université de Montpellier, CNRS, UMR 5048, Centre de Biochimie Structurale, Montpellier, France; Department of Cell and Developmental Biology, Weill Cornell Medical College of Cornell University, New York, NY USA; Inserm, U1004, Villejuif, France; Institut André Lwoff, Université Paris 11, Villejuif, France; Centre de Biochimie Structurale, 29, rue de Navacelles, 34090 Montpellier, France

**Keywords:** Single molecule tracking, Membrane, Diffusion, Trajectory, Neural network

## Abstract

**Background:**

Single particle tracking (SPT) is nowadays one of the most popular technique to probe spatio-temporal dynamics of proteins diffusing within the plasma membrane. Indeed membrane components of eukaryotic cells are very dynamic molecules and can diffuse according to different motion modes. Trajectories are often reconstructed frame-by-frame and dynamic properties often evaluated using mean square displacement (MSD) analysis. However, to get statistically significant results in tracking experiments, analysis of a large number of trajectories is required and new methods facilitating this analysis are still needed.

**Results:**

In this study we developed a new algorithm based on back-propagation neural network (BPNN) and MSD analysis using a sliding window. The neural network was trained and cross validated with short synthetic trajectories. For simulated and experimental data, the algorithm was shown to accurately discriminate between Brownian, confined and directed diffusion modes within one trajectory, the 3 main of diffusion encountered for proteins diffusing within biological membranes. It does not require a minimum number of observed particle displacements within the trajectory to infer the presence of multiple motion states. The size of the sliding window was small enough to measure local behavior and to detect switches between different diffusion modes for segments as short as 20 frames. It also provides quantitative information from each segment of these trajectories. Besides its ability to detect switches between 3 modes of diffusion, this algorithm is able to analyze simultaneously hundreds of trajectories with a short computational time.

**Conclusion:**

This new algorithm, implemented in powerful and handy software, provides a new conceptual and versatile tool, to accurately analyze the dynamic behavior of membrane components.

**Electronic supplementary material:**

The online version of this article (doi:10.1186/s12859-016-1064-z) contains supplementary material, which is available to authorized users.

## Background

Diffusion and partition of components of the plasma membrane into specialized areas, especially their lateral segregation and organization into micro or nanodomains, are key events in cell function. Different domains involving lipid-protein-protein interactions have been identified at the cell surface and most of them are enriched in specific lipids such as sphingolipids and cholesterol. It has been proposed that these lipids create peculiar physical properties within membranes, which are known as liquid ordered phase (reviewed in [1, 2]). Proteins anchored to the actin cytoskeleton could also be a barrier confining membrane components in small regions of plasma membrane [3]. In order to better understand the molecular mechanisms underlying this lateral segregation, the diffusive behavior of membrane components has been investigated in living cells using techniques such as fluorescence recovery after photobleaching (FRAP), fluorescence correlation spectroscopy (FCS), and single particle tracking (SPT) (reviewed in [4]). It has been shown that diffusion of membrane proteins in cell membranes is not limited to pure Brownian diffusion. Indeed, two other modes of motion have been so far identified within membranes, namely directed and confined, that includes tethered diffusion or diffusion in the presence of obstacles [5]. In addition numerous studies have highlighted that single molecules could present complex behavior, namely switching between the different modes of diffusion described above. For instance, it was early described by Jacobson’s group that the glycosylphosphatidylinositol (GPI)-anchored protein Thy1 could be transiently confined (here alternating between free diffusion and confinement) in specific areas identified as raft microdomains [6]. More recently, a similar behavior was observed with another GPI-anchored proteins (CD59) that can be transiently trapped in confinement zones named STALL (Stimulation-Induced Temporary Arrest of Lateral Diffusion) [7]. A combination of confined and Brownian diffusion modes within a trajectory has also been observed for transmembrane proteins such as tetraspanins [5], the epidermal growth factor (EGF) receptor [8] and the cystic fibrosis transmembrane conductance regulator (CFTR) channel [9]. Also transient directed motion was observed for gamma amino butyric acid (GABA) receptors in nerve growth cones [10].

SPT that consists in visualizing diffusing single molecules and frame-by-frame reconstruction of their trajectories is especially relevant to probe the dynamics of heterogeneous systems such as biological membranes or, more generally, diffusion and transport of molecules or cargos in cells. SPT can locate each particle with sub-diffraction resolution by reshaping the optical point spread function and measure its dynamics, instead of an ensemble average. Nowadays, tracking is mainly performed using fluorescent proteins or chemical dyes. In the case of single molecule tracking, very stable fluorescent probes are required such as the organic dye Atto647N (e.g. [11, 12]) or quantum dots [13]. Labeled molecules are then detected using high numerical aperture objectives and highly sensitive and rapid cameras. Using such strategy, it is now possible to track fluorescent single molecules within membranes of living cells and to analyze their behavior, i.e. diffusion coefficient, velocity and motion modes. Analysis of diffusion in SPT experiments is generally performed by connecting the position of fluorescent dots at different time points into a trajectory and by plotting the mean-square displacement (*MSD*) < *r*^*2*^ > versus time lag. From this plot, one can discriminate between Brownian, confined or directed trajectories (reviewed in [14]). However, it is also important to identify different modes of diffusion within a trajectory and three main types of methodologies have been developed so far. The first one is based on *MSD* analysis. Jacobson’s group has early created an algorithm, which is based on the determination of a confinement index that corresponds to the probability that a given protein would stay in a region for a period of time [6] (see also an improvement of this method in [15]). An algorithm to detect transient directed motion has also been developed by Dahan’s group by calculating a speed correlation index describing the high temporal correlations of the speed in directed motions compared to Brownian motion [10]. In both techniques a sliding time window is used to achieve a local analysis. The second methodology is based on Bayesian analysis. Masson’s group developed a Bayesian decision tree for the classification of the mode of motion of single molecule trajectories, especially suitable to discriminate between Brownian and confined trajectories [16] whereas Bathe’s group applied Bayesian model selection to hidden Markov modeling to infer transient transport states of mRNA-protein complexes often displaying trajectories alternating directed and Brownian trajectories [17]. The third methodology is based on a supervised support vector classification (called SVM) suitable to segment a trajectory into different motion modes including directed and confined behaviors, without sliding windows [18]. Another way to describe the heterogeneity of plasma membrane is to evaluate the anomalous sub-diffusion, a hindered diffusion in which the hindrances change the actual form of the time dependence, not just the numerical value of the diffusion coefficient [19]. Anomalous diffusion is described by the power law < *r*^*2*^ > ~Dt^α^ where α equal to 1 for Brownian diffusion, <1 for sub-diffusion and >1 for super-diffusion. The presence of multiple diffusion regimes within a trajectory can also be determined by analyzing the probability distribution of square displacement (*PDSD*) [20–22]. In this case, *PDSD* describes the probability that a particle will be found within a circle of radius (*r*) at time (*t*). With the two last methods, it is impossible to know when and where the change in the diffusion mode occurs.

**Fig. 1:**
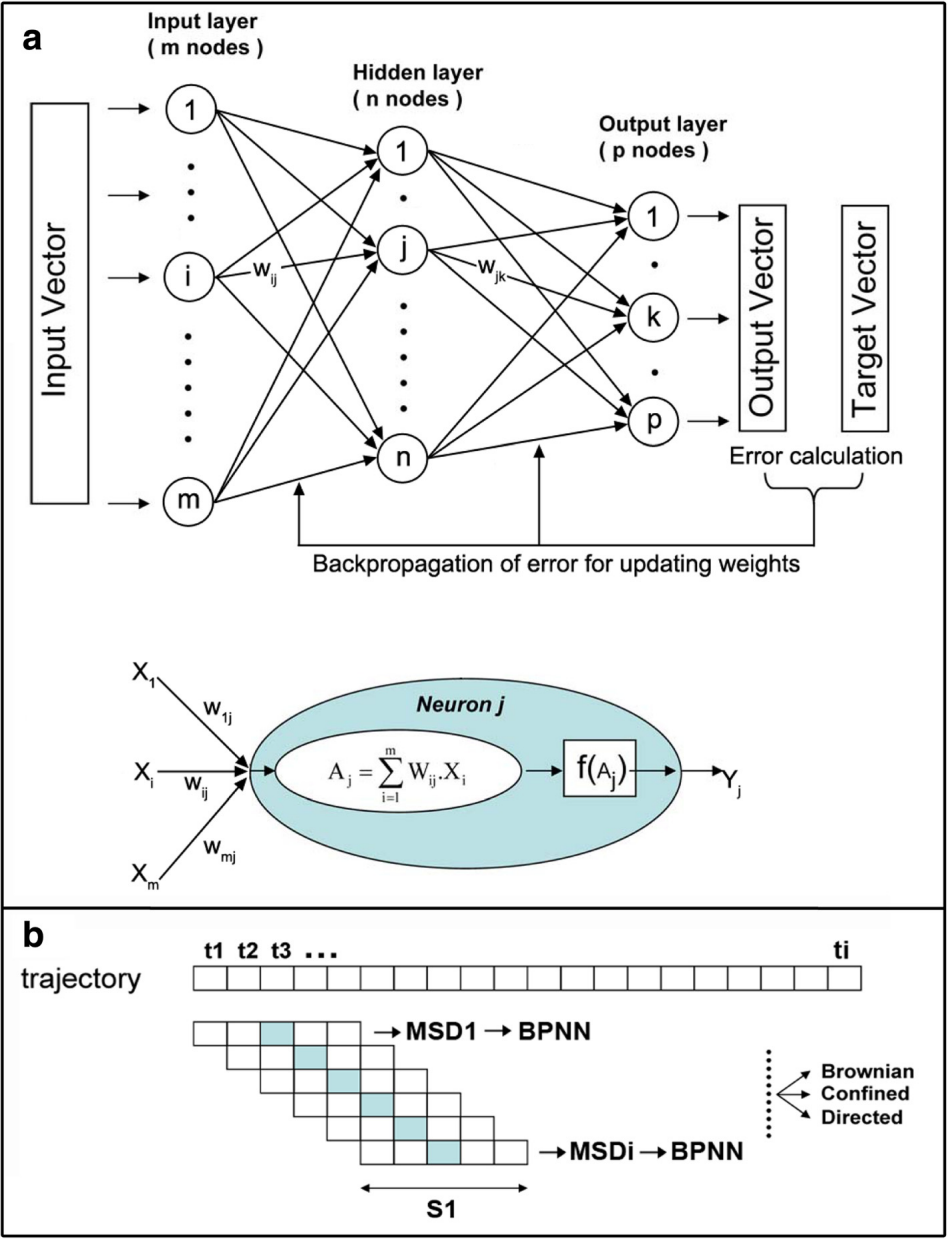
Architecture of the neural networks. **a** Upper panel: schematic view of the three-layer back propagation neural network (BPNN). Each input value is passed through the neural network. The output value is compared with the desired target output, an error is computed and this error is propagated backward through the network to each node. Lower panel: graphical representation of the model neuron *j* or threshold unit. The threshold unit receives input, called *Xi*, from m other units. The associated weight is called *W*_*i*_. The total input *A*_*j*_ is the sum over all inputs. The activation function *f (A*_*j*_ *)* of the neuron is a sigmoid and *Y*_*j*_ is the output of the neuron. **b** Schematic diagram of the algorithm used to detect 2D diffusion modes within a trajectory. The trajectory is split in overlapping segments of length S1 using a sliding window. The MSD curve is calculated for each segment, normalized, presented to the neural network and classified according to three main diffusion modes (Brownian, directed or confined). A score (output value) is obtained for each frame of the movie and attributed to all the frames of the sliding window

In this paper we present a new approach to automatically discriminate between different modes of membrane diffusion within the same trajectory using back-propagation neural network (BPNN). BPNN is the most widely used type of artificial neural networks (reviewed in [23]). It typically consists in many simple processing elements called neurons, which are grouped in layers. All nodes of a layer are connected to all the nodes in the adjacent layers by interconnections called synapses (Fig. 1a). Here BPNN was trained using simulated Brownian, confined, or directed trajectories to determine the motion modes of single molecule within a trajectory thanks to a sliding window. Validation of the algorithm in identifying different motion modes was achieved using synthetic data. The algorithm was then tested with trajectories recorded in living cells, especially a transmembrane protein diffusing within the plasma membrane.

## Results

### A. Backpropagation Neural Networks Architecture and algorithm

BPNN was created with one hidden layer between input and output units (Fig. 1). All nodes of a layer were connected to all the nodes in the adjacent layers. The BPNN included two working phases, the learning and the recall phase. During the learning phase, known data sets were used as a training signal in input and output layers. The first operation for the learning phase is the feed-forward operation. During this operation, each input neuron receives an input signal and broadcasts this signal to the connected neurons in the hidden layer. Each neuron in the hidden layer computes its activation and sends the result to the output neuron. Mathematically, a 3-layer BPNN with *m, n*, and *p,* the number of input, hidden and output nodes, respectively, is based on the following equation:

1 $$ {O}_k=f\left({\displaystyle \sum_{j=1}^n{W}_{jk}}\times f\left({\displaystyle \sum_{i=1}^m{W}_{ij}{X}_i}\right)\right) $$

*O*_*k*_ is the calculated output of the *k*^th^ neuron in the output layer, *X*_*i*_ the input values of the network, *W*_*ij*_ the connection weight from node *i* (input layer) to node *j* (hidden layer), *W*_*jk*_ the connection weight from node *j* (hidden layer) to node *k* (output layer), *f* is the activation function of the neuron which is classically a sigmoid function as defined in Eq. (2).

2 $$ f(x)=\frac{1}{1+{e}^{-x}} $$

Targeted outputs from each training pattern are compared with the actual activation level of the output units and the difference between the two determines the system error (Eq. 3).

3 $$ E=0.5{\displaystyle \sum_{k=1}^p{\left({O}_k-{T}_k\right)}^2} $$

*p* is the number of output neurons, *O*_*k*_ and *T*_*k*_ are the calculated output and the target output, respectively, of the *k*^th^ neuron in the output layer.

The second operation of the learning phase is the backward pass in which a gradient descent method is used to minimize the error in the training set. Starting from the output layer, the error is backward propagated through the network, layer by layer, by recursively computing the local gradient error of each neuron. The connection weight is then changed in proportion to the negative of an error derivative using the following equation:

4 $$ \varDelta {W}_{i,j}\left(t+1\right)=-\eta \frac{\partial E}{\partial {W}_{i,j}} + \alpha \varDelta {W}_{i,j}(t) $$

*ΔW*_*i*,*j*_(*t* + 1) is the weight increment minimizing E between the *j*^th^ neuron and the *i*^th^ neuron at the (t + 1) ^th^ iteration. *η* is the learning rate and *α* is the momentum parameter. *α* is chosen between 0 and 1, typically 0.9, a value allowing high learning rates [24]. Using a momentum term is the simplest method to avoid oscillation problems when searching the minimum value of the error surface. This forward-backward process is repeated for each input signal.

The BPNN is trained by repeatedly presenting a series of input/output pattern sets to minimize the mean squared error (MSE) (Eq. 5):

5 $$ MSE=\frac{1}{n_xp}{\displaystyle \sum_{l=1}^n}{{\displaystyle \sum_{k=1}^p\left({T}_{lk}-{O}_{lk}\right)}}^2 $$

*n* is the number of input vector and *p* is the number of output neurons. *O*_*lk*_ and *T*_*lk*_ respectively denote the calculated output and the desired output of the *k*^th^ neuron when the input vector l is applied to the network. The recall phase is performed in one pass using the weight obtained in the learning phase.

The input signal of this BPNN is the normalized mean square displacement (*MSD*) of a trajectory as a function of time. *MSD* analysis is one of the most widely used approaches to extract reliable values of the diffusion coefficient *D* and consists in plotting the *MSD* versus time lag (*δt*) according to the equation:

6 $$ MSD\left(n\delta t\right)=\frac{1}{N-1-n}{\displaystyle \sum_{j=1}^{N-1-n}\left\{{\displaystyle {\left[x\left(j\delta t+n\delta t\right)-x\left(j\delta t\right)\right]}^2}+{\displaystyle {\left[y\left(j\delta t+n\delta t\right)-y\left(j\delta t\right)\right]}^2}\right\}} $$

where *δt* is the time interval between two successive frames, *x (t)* and *y (t)* are the particle coordinates at time *t*, *N* is the total number of frames, and *n* is the number of time intervals [25] (for more details, see the Methods section). For Brownian motion, the MSD curve increases linearly with time increment *δt*. The curve exhibits an upward or downward curvature for directed or confined motions, respectively. In our system, the 3 types of movements described above are discriminated by analyzing the trajectory with the algorithm using a sliding window with a size *S1* (Fig. 1b). A three-layer BPNN with three output nodes was constructed, one for each motion type, namely Brownian, directed and confined. We chose one hidden layer with five hidden nodes (see more details in the Materials and Methods section). Input variables were normalized and scaled into values in the range of 0.0–1.0). Three output values are assigned to each motion mode and normalized.

### Training procedure and validation sets

The network was trained using data sets of simulated trajectories. One thousand trajectories of 3.1 s duration (31 frames, the size of the sliding window in this example) were simulated for each type of diffusion mode with a 100 ms time increment. For confined diffusion, the particle displays a Brownian diffusion in a constrained area with a variable diameter value *L*. For directed diffusion, a term of constant drift velocity *V* in one direction all along the trajectory is added to the Brownian movement. One part of the data sets is used for training, the other one for cross validating with different parameters (see Material and Methods).

**Fig. 2:**
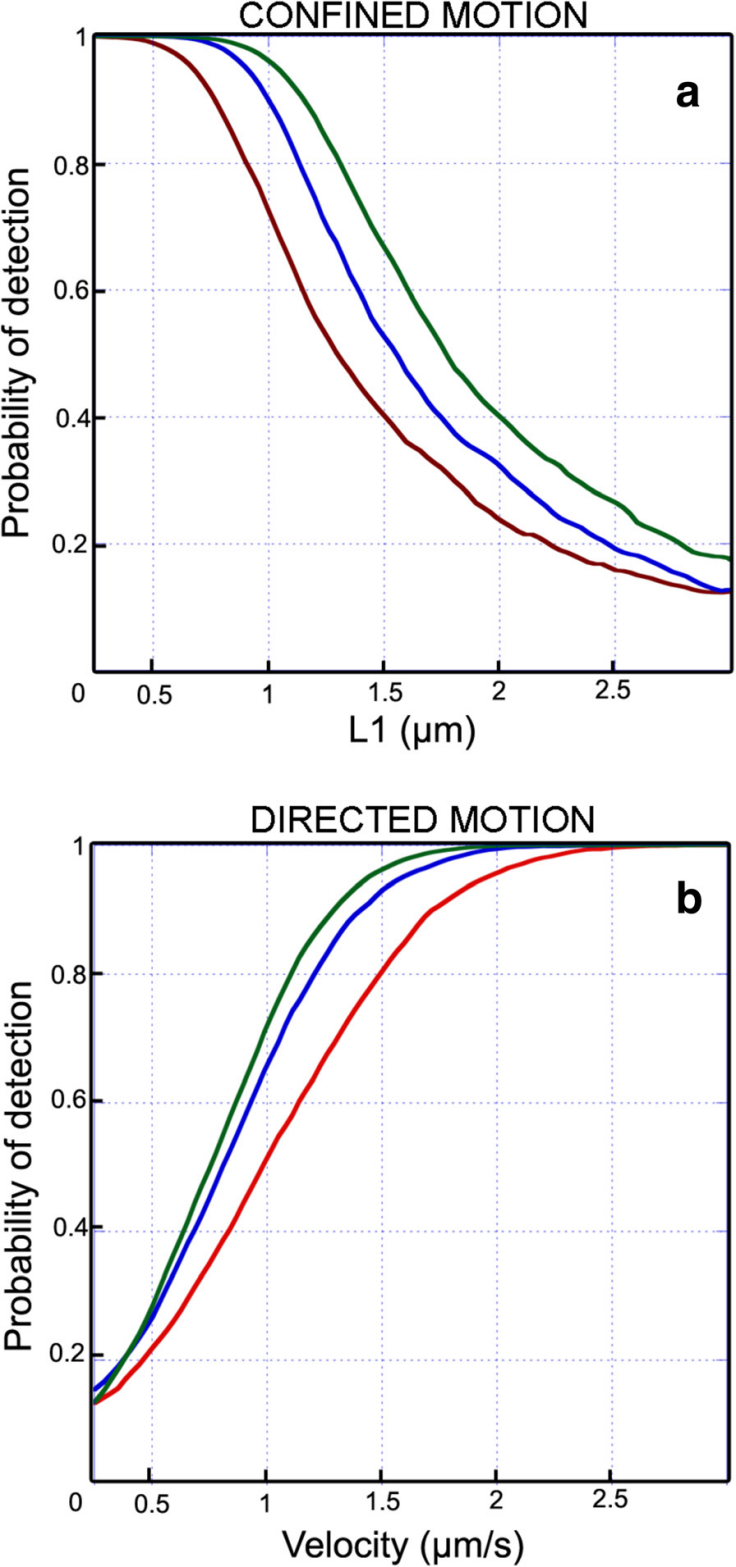
Percentage of detection of confined and directed diffusion modes as a function of the size of the sliding window S1. The probability of detection of confinement or directed motion in simulated trajectories was calculated for different length of segment used to calculate the *MSD* (S1 equal to 21 (red), 31 (blue) or 41 (green) frames). The probability of detection of confinement in trajectories is expressed as a function of the diameter confinement *L* (**a**) whereas the probability of detection of directed motion is expressed as a function of the velocity (**b**)

The performance of a trained BPNN was first evaluated using new naive simulated patterns and by computing the percentage error between calculated output and expected values (probability of detection). It increased for a given diffusion coefficient when the confinement size decreased for confined trajectories (Fig. 2a) or when the velocity increases for directed trajectories (Fig. 2b). The learning procedure was performed with 3 different datasets of trajectories of 2.1, 3.1 and 4.1 s durations. Under our conditions, a similar probability of detection of directed diffusion modes was obtained for 31 and 41 frames. The size of the sliding window was therefore fixed to 31 frames (blue curve in Fig. 2), a value at which the probability of detection of 1 μm diameter confinement (a reasonable size of confinement in cell membranes) is still possible and obviously allows detecting smaller segments as compared to 41 frames. Considering 2 directed trajectories *T*_*0*_ and *T*_*1*_ with respective diffusion coefficient *D*_*0*_ and *D*_*1*_ and velocity *V*_*0*_ and *V*_*1*_, the normalized MSD curves will be similar if:

7 $$ {V}_1={V}_0\sqrt{\frac{D_1}{D_0}} $$

Similarly the normalized MSD curves will be similar between 2 confined trajectories *T*_*0*_ and *T*_*1*_ with respective diffusion coefficient *D*_*0*_ and *D*_*1*_ and confinement diameter *L*_*0*_ and *L*_*1*_, if:

8 $$ {L}_1={L}_0\sqrt{\frac{D_1}{D_0}} $$

### Determination of the detection threshold of confined and directed motion within a trajectory

**Fig. 3:**
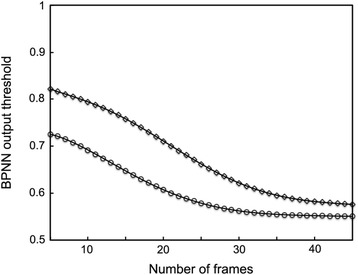
Determination of the detection threshold of confined and directed diffusion modes. The BPNN output thresholds for confined diffusion mode *Y*_*C*_ (empty circles) and directed diffusion mode *Y*_*D*_ (empty squares) were calculated for confined diffusion (*Y*_*C*_) mode (empty circles) and directed diffusion mode (empty squares) by plotting as a function of the number of consecutive frames (the length of detected segment) the BPNN output for which the neural network falsely indicated, in 5 % of the cases, a confined or directed motion for a Brownian trajectory

**Fig. 4:**
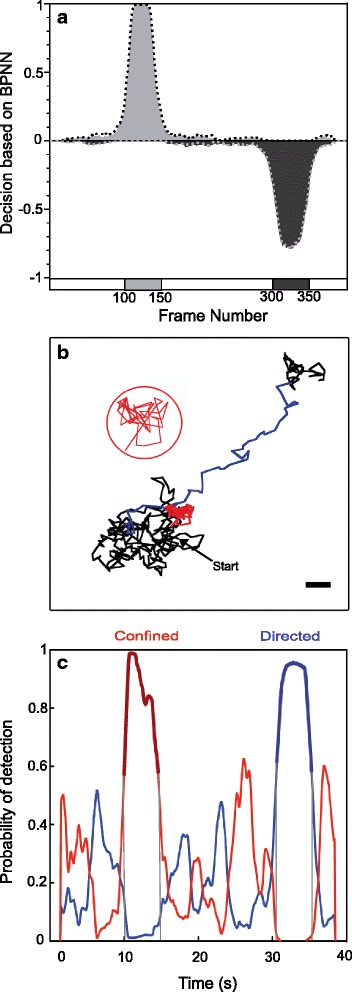
Analysis of the motion modes within synthetic trajectories. **a** Detection probability using BPNN. 200 trajectories of 400 frames including one directed motion segment with 1.2 μm/s velocity and one confinement segment with 1 μm diameter were analyzed with BPPN (confined in light grey, directed in dark grey; *D* = 0.25 μm^2^/s, integration time = 100 ms; each 50 frames segment is always localized at the same position). The percentage of decision based on BPNN corresponds to the number of positive decision for a specific motion mode detected for a given frame over 200 trajectories and normalized to 1 or -1 for confined or directed trajectories, respectively (black, confined trajectories; grey, directed trajectories). The algorithm was also tested with a 30 nm localization noise (dotted lines in the graph). **b** The upper panel shows a synthetic trajectory of 40 s (400 frames) including a transient confinement (from 10 to 15 s) with a diameter of 1 μm (red trace, zoomed in the red circle) and a transient directed motion (30 to 35 s) with a velocity *V* = 1.2 μm/s (blue trace). The Brownian part is in black. The diffusion coefficient *D* is 0.25 μm^2^/s and the integration time 100 ms. Scale bar, 1 μm. **c** The Lower panel is the plot of the probability of detection of motion mode (BPNN output values) as a function of the duration time of the trajectory for confined (red) or directed (blue) motion. Confined (bold red trace) and directed (bold blue trace) segments are respectively detected between 10.0 and 14.6 s and between 30.6 and 35.3 s as shown by grey lines. The detection threshold values correspond to a probability of detection with a 95 % confidence (see Fig. 4). The calculated diameter of confinement is 1.06 μm

The 31 frames sliding window described above is used to split the trajectory in different segments and is positioned at the point *i = (S*_*1*_*-1)/2* of the trajectory in order to define a first segment containing the first 31 points. The *MSD* curve is calculated for this 31 frames trajectory and is presented as an input to the neural network after normalization. The 3 output values of the neural network *O*_*B*_, *O*_*C*_ and *O*_*D*_ correspond to the probability of the particle to diffuse according to one of the 3 diffusion modes (Brownian, confined and directed, respectively). These 3 values are attributed for each frame of the sliding windows. The sliding window is then translated to the point *i + 1* in order to analyze the next segment of the trajectory and the procedure is repeated up to the point *i + n = N-[(S*_*1*_*-1)/2]*. For each frame of the trajectory, the average of output values (*O*_*B*_*, O*_*C*_*and O*_*D*_) corresponding to the probability of assignment of one of the 3 diffusion modes of each frame during BPNN analysis, are plotted as a function of time (several output values are provided for a given frame due to the sliding windows). However, statistically, some pure Brownian trajectories can transiently display a behavior that is similar to confined or directed ones. Therefore, we determined a threshold value in order to distinguish the true confined or directed part of the trajectory from that due to Brownian fluctuations. To do so, 100 Brownian trajectories of 1000 frames were generated (increment of time, 100 ms; D, 0.25 μm^2^/s) and analyzed. We then plotted the BPNN output values threshold Y_C_ (or Y_D_) as a function of the number of consecutive frames (from 5 to 45 frames) for which the percentage of falsely attributed as confined (or directed) segments by the neural network did not excess 5 % of the total number of frames of the Brownian trajectory (95 % confidence) (Fig. 3). These thresholds are used to identify the segments that are confined or directed within a trajectory (see an example in Fig. 4c).

### B. Validation of the algorithm

The algorithm was first validated using 200 synthetic trajectories of 400 frames, containing one 50 frames segment of both confinement and directed motion (1 μm for the confinement diameter and 1.2 μm/s for the velocity, respectively; 100 ms for the time increment and 0.25 μm^2^/s for the diffusion coefficient), tested with or without a 30 nm localization noise. The percentage of decision based on BPNN corresponds to the number of positive decision for a specific motion mode detected for a given frame and normalized to 1 or -1 for confined or directed trajectories, respectively (Fig. 4a). Confined and directed segments were detected by the BPNN with good accuracy (99 and 78 % detection of the confined and directed segments within a 50 frames segment). An example of trajectory is shown in Fig. 4b and c. Duration of confinement and directed segments was slightly underestimated by the BPNN, respectively 4.6 s and 4.7 s instead of 5 s. Data from part of the trajectory identified as directed or confined were respectively fitted with the equations 2 and 3 described in the Methods section. The confinement diameter and the velocity were estimated to be 1.06 μm and 1.2 μm/s, respectively. These values are in good agreement with the parameters of the simulation. As expected, apparent diffusion coefficients from Brownian segments were estimated around 0.25 μm^2^/s. No changes in the BPNN-based decision were observed when adding 30 nm positioning noise.

We also evaluated the capacity of BPNN to detect small segments within a trajectory. 200 simulated trajectories of 300 frames containing confined segments of various lengths were created and the probability of detection named decision based on BPNN was calculated as a function of the segment length. A similar process was used for directed motion mode (Additional file 1: Figure S1). Our results indicate that our algorithm is able to detect 99 and 90 % of the confined or directed motion modes within a 40 frames segment, respectively. These values dropped down to 74 and 72 % for 30 frames segments and to 27 and 43 % for a 20 frames segment. The percentage of detection dropped below 10 % when the length of the segment is smaller than 10 frames. It is important to indicate that this evaluation has been performed with diffusion parameters close to those often encountered within biological membranes (100 ms time integration, a 0.25 μm^2^/s diffusion coefficient of the particles, velocity ranging from 1 to 3 μm/s for directed trajectories and confinement diameter from 0.5 to 1.2 μm). The percentage of detection could be largely improved for a higher velocity (60 % and 100 % detection for *V* = 4 μm/s using 10 and 20 frames, respectively; data not shown). Similarly decreasing the sliding window improves the detection accuracy for confined trajectories. Our objective here was to provide as a proof of concept a tool with efficient detection of both directed and confined segments within the same trajectory.

### C. Comparison with other methods

As mentioned in introduction, several algorithms have been developed to segment different types of motion within trajectories. We then compared our BPNN-based method to 3 freely available algorithms. Two methods are based on Bayesian analysis and specialized in the detection of confined (Bayesian Information Criteria named BIC [16]) or directed (Hidden Markov Modeling (HMM)-Bayes [17]) segments within a trajectory. The third method is a machine learning method based on trajectory segmentation using supervised support vector (SVM) classification [18]. The comparison was performed using setting parameters and simulated trajectories similar to what is described above, namely trajectories of 300 frames including one directed motion segment and one confinement segment always localized at the same position, both with a length of 50 frames, the velocity randomly ranging from 1 to 3 μm/s for directed motion and one confinement segment with diameters ranging from 0.5 and 1.2 μm for confined motion (Additional file 2: Figure S2). Under theses conditions, the four methods were found very specific with detection accuracy larger than 95 % (the SVM method was the most specific with 99.9 % detection accuracy). Larger differences were observed in terms of sensitivity and our algorithm was able to respectively detect 75.3 and 83.1 % of confined and directed 50 frames segments within a 300 frames trajectory (Additional file 2: Figure S2). We also evaluated the time of calculation that is important to take into account since SPT requires a large sampling to get significant results. Most likely because our algorithm was developed in the Visual C++ environment, the computational time was very low, typically less than 1 s for analyzing a 300 frames trajectory (*D*, 0.25 μm^2^/s; integration time, 100 ms) using a PC Windows 7 Intel (R) Core 5TM) i7-2640 M CPU 2.8 GHz (data not shown).

### D. Segmentation of real trajectories

**Fig. 5:**
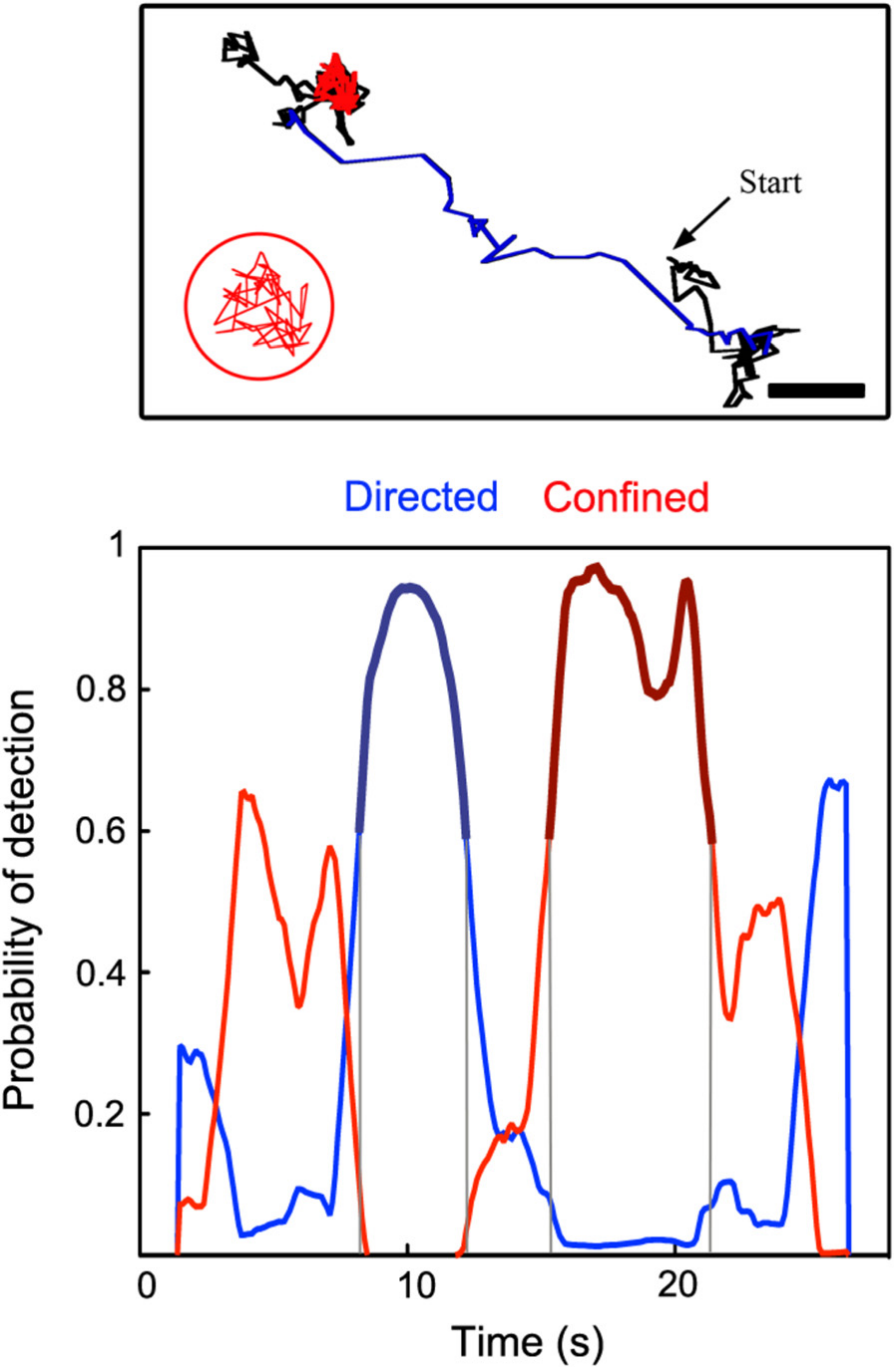
Analysis of the motion modes within a real trajectory of a Moloney Murine Leukemia virus (MLV) particle in HEK cells. The upper panel shows the trajectory of a MLV particle containing YFP-tagged Gag proteins recorded in infected 293 HEK cells, tracked using TIRF microscopy and recorded at a 100 ms integration time. The confinement area is zoomed in the red circle. The lower panel is the plot of the probability of detection of motion mode (BPNN output values) as a function of the duration time of the trajectory. A zone of confinement is detected during 6.1 s (bold red trace) with a diffusion coefficient of 0.01 μm^2^/s and a diameter of 170 nm. Directed motion is indicated during 4 s with a diffusion coefficient of 0.012 μm^2^/s and a velocity of 0.38 μm/s. Scale bar, 500 nm

**Fig. 6:**
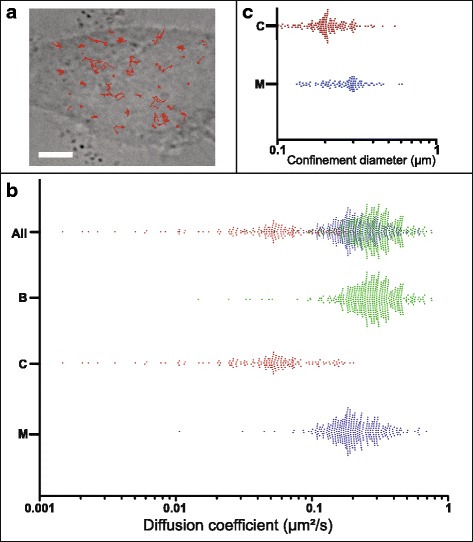
Single molecule tracking of the tetraspanin CD9 in HeLa cells. The (**a**) panel is a DIC image of a single HeLa cell and the red lines represents some recorded trajectories of single molecules (the scale bar is 5 μm). The lower panel (**b**) represents the scatter plots of CD9 apparent diffusion coefficients calculated from the MSD analysis of 1000 trajectories in HeLa cells. Each point represents one trajectory and the diffusion behavior, which has been determined using the BPNN neural network, is indicated with a color code (red, Confined named (**c**) green, Brownian named (**b**) blue, mixed named M). The upper scatter plot corresponds to the totality of the trajectories (named all). Panel (**c**) shows the confinement diameter calculated from the confinement segments identified using BPNN for confined and mixed trajectories

The algorithm was then used to segment trajectories reconstructed from movies recorded in living cells. The BPNN was used in the context of the tracking of YFP-labeled Moloney Murine Leukemia viruses (MLV) in human embryonic kidney (HEK) cells using total internal reflection fluorescence (TIRF) microscopy (see the Methods section). The behavior of such a viral particle can be Brownian, confined when trapped within membrane of host cells or directed when the particle is exported from the cytoplasm to the plasma membrane [26, 27]. An example of such a complex trajectory alternating between different motion modes is shown in Fig. 5a and the analysis by the neural network in Fig. 5b. The algorithm detects three different motion modes within the trajectory. A confinement was detected during 6.1 s in a zone of 170 nm diameter, a size in good agreement with a previous report for murine polyoma virus-like particles [28]. Directed motion was also observed during 4 s with a velocity of 0.38 μm/s that compares well with that previously reported for MLV (0.57 μm/s in [29]). The rest of the trajectory was Brownian. We also tested the BPNN algorithm by analyzing a set of trajectories of the tetraspanin CD9 recorded in HeLa cells (Fig. 6). This molecule is a transmembrane protein expressed in the plasma membrane that has been demonstrated to diffuse mostly in a Brownian mode but can be transiently or permanently confined in membrane areas enriched in tetraspanins and their partners (reviewed in [30]). Trajectories including transient confinement of CD9 molecules are called “mixed trajectories” because of the combination of Brownian and confined behavior. Apparent diffusion coefficient of 1000 CD9 molecules was first determined using the first points (D_1-4_) of the plot of the MSD as a function of time (upper part of the scatter plot in Fig. 6, each dot representing one molecule) and the mean value of CD9 apparent diffusion coefficient (0.23 ± 0.04 μm^2^/s) was similar to that previously reported in the plasma membrane of HeLa cells [31]. Interestingly the BPNN was able to directly detect mixed trajectories (blue dots in the scatter plot in Fig. 6), in addition to pure Brownian (green) and pure confined (red) trajectories (38.5, 45.6 and 15.9 % of the total trajectories, respectively). These percentages also compared well with those described in [31]. Similarly to what is described in Fig. 6, the BPNN provided the number of frames of each identified segments and the corresponding apparent diffusion coefficient (D_1-4_). The diameter of confinement associated to these segments was calculated from MSD analysis and, in agreement with a previous report, the diameter of confinement was smaller for pure confined trajectories as compared to the one of confined segment in mixed trajectories (215 ± 68 nm versus 273 ± 89 nm).

## Discussion

In this work we propose a new approach to identify and automatically characterize the diffusion behavior of single molecules encountered within biological membranes. The novel algorithm we have developed is based on artificial neural network analysis that has already been applied in biology, e.g. to predict protein secondary structures [32]. Such a computational approach is especially suitable to solve complex behavior with non-linear relationships between independent and dependent variables. We demonstrate here that it can be applied to probe the heterogeneity encountered within trajectories of membrane components of eukaryotic cells by segmenting trajectories into different modes of diffusion with a good accuracy. Based on MSD analysis, the algorithm is able to accurately discriminate between Brownian, confined and directed diffusion over a wide range of confinement areas and velocity and it is therefore possible to detect transition between these 3 modes. This is especially interesting in the light of observations showing that diffusing molecules or particles often display a complex behavior rather than a single motion mode. Such an analysis can provide important information about the mechanisms of membrane partitioning in living cells, especially when combining SPT and ensemble labeling (see one example in [11]).

The new algorithm is based on a BPNN trained with short synthetic trajectories of particles displaying Brownian, confined or directed motion modes. We intentionally chose sets of synthetic trajectories with diffusion parameters close to the values mainly described in the literature for proteins diffusing within biological membranes using single molecule fluorescence microscopy. The algorithm could be eventually further developed by training the BPNN with a larger range of values for the different modes of diffusion, but could also included new mode of diffusion such as slow and fast drifting motion (this detection is already available with the SVM method). It could probably be extended to the analysis based on anomalous diffusion.

Similarly to other trajectory segmentation-based algorithms for analyzing motion modes [8, 18, 33, 34], detection was performed using a sliding window allowing detection of temporal changes in the mode of motion within a trajectory. Here, we applied a sliding window of 31 frames corresponding to the length of synthetic trajectories used for BPNN training. We chose a segment length small enough to measure local behavior but large enough to get sufficiently accurate output values. Under these conditions, it was possible to confidently identify motion modes for segment sizes as short as 20 frames. This value compared well with other algorithms that do not use a sliding window, e.g. the segmentation of trajectories based on based on supervised support vector classification that requires a minimum segment length of 25 frames to get 60 % detection rate [18]. Some algorithms are able to detect smaller segments such as the HMM-Bayes algorithm that is very accurate in detecting directed diffusion segments of 5 frames. However it requires a minimum number of observed particle displacements within the trajectory to infer the presence of multiple motion states [17] whereas our algorithm does not require prior information, a very important feature since the diffusion-state switching occurrences is a stochastic process and that the trajectories recorded with organic dyes are generally short due to low photo-stability. Under the experimental conditions tested in this paper, which mimic the plasma membrane of eukaryotic cells, our method was found very sensitive, specific and versatile, able to accurately detect both confined and directed segments within trajectories. It appears to be very competitive in regards to the results obtained with 3 other methods already freely available, at least in the conditions tested in this study. In addition, the computational time required by our algorithm is very low, a very important feature since SPT requires the analysis of several hundreds of trajectories. This is partly due to its implementation in Visual C++, which is stable even for high load. The BPNN algorithm could be applied to high-throughput analysis or screens. Another interesting property lies in its absence of sensitivity to the noise positioning (at least for 30 nm).

## Conclusion

We demonstrated in this study that our algorithm provides a new conceptual, versatile and useful tool in dissecting complex trajectories, identifying different motion modes and providing diffusion-associated parameters of the identified segments. It requires the adjustment of a minimal set of parameters. Combined with our homemade software named PaTrack, which is freely available on the website of the laboratory [35], our algorithm can accurately analyze membrane dynamics of single molecules or larger assemblies, such as viruses [36].

## Methods

### Generation of synthetic trajectories

Brownian diffusion in two dimensions was simulated as a random walk process. *X* and *Y* coordinates of each particle were sampled step by step from normal distribution with zero mean and standard deviation of $\sqrt{2D\tau }$ where *D* is the diffusion coefficient and *τ* the time interval between 2 frames. For confined trajectories, the particles freely diffuse inside a domain with a diameter *L*. Directed trajectories were constructed by adding a velocity term *V*.

### Learning and cross validation of BPNN

We first built a BPNN with one hidden layer and used using cross validation to evaluate over fitting and to determine the optimal number of hidden neurons. 3000 synthetic trajectories of 31 frames (1000 for each mode) were created and split in 2 sets, one for learning (3 × 700 trajectories) and one for cross validating (3 × 300 trajectories). Confinement diameter randomly varied between 0.25 and 2 μm in confined trajectories and *v* randomly varied between 0.5 and 1.5 μm/s. Diffusion coefficient and time intervals were 0.25 μm^2^/s and 100 ms, respectively. The MSD curve was calculated and used as a BPNN input signal. During the learning phase the mean square error (MSE) was regularly calculated for the set of trajectories used for cross validation. Since back propagation uses a gradient-descent procedure, the error for the learning process was decreasing with the number of iterations and the training was stopped when the network started to overfit the data, which corresponds to a MSE increase for the cross validation set [23]. The learning phase was performed for different number of hidden neurons and the combination given the lowest MSE for the cross validation was chosen (set to 5 here). Adding another hidden layer did not improve the performance of the neural network.

### BPNN validation

In order to validate the BPNN algorithm, heterogeneous synthetic trajectories were created by assembling different segments with different motion modes (the properties of synthetic trajectories are mentioned in the results section). When mentioned, a static positioning noise *P*_*n*_ (localization error) of 30 nm was added to the trajectory by an additional displacement taken from a Gaussian distribution with standard deviation 2*P*_*n*_ with an angle randomly distributed over [0,2π]. This Gaussian noise models all sources of noise, i.e. Poissonian photon shot noise due to signal and fluorescence background, detector noise, pixelization effects, and error of the localization algorithm using a Gaussian representation [16].

### Tracking procedure

All movies or simulated data were analyzed using homemade software named “PaTrack” developed in the Visual C++ environment (Microsoft, Washington, USA), in which the BPNN-based algorithm has been implemented. This software is available online using the link “http://www.cbs.cnrs.fr/index.php/en/platforms-facilities/softwares” where it can be downloaded with the complete instructions manual. Single molecule tracking was based on point spread function (PSF) fitting. MSD was computed according to Equation 7. Apparent diffusion coefficient values were determined from a linear fit between the first and fourth points D_1-4_ of the MSD versus time lag as previously reported [3]. Once the associated motion modes were identified by the BPNN, segments were fitted with the following equation: *MSD (Δt) = 4DΔt* for a simple diffusion, *MSD (Δt) = 4DΔt + V*^*2*^*(Δt)*^*2*^ for directed diffusion and *MSD (Δt) = (1/3) L*^*2*^*[1–e (-12DΔt/L*^*2*^*)]* for confined diffusion, where *V* is the constant drift velocity, *L*^*2*^ the area of the confined region and *D* the diffusion coefficient. Data analysis of the different particles is performed step by step.

### Tracking in living cells

SPT experiments were carried out as previously described [11, 31]. For CD9, HeLa cells were plated on 25-mm Ø glass coverslips and incubated in culture medium at 37 °C for 10 min with Atto647N-labeled Fab fragments of the anti-CD9 mAb, SYB-1 [37]. A home made objective-type TIRF setup allowing multicolor single molecule imaging and equipped with an αPlan Fluor 100×/1,45 NA objective (Zeiss, Le Peck, France Brattleboro, VT) was used. All the experiments were done with a 100 ms integration time. For MLV, human embryonic kidney HEK293 cells were transfected with the MLV YFP-GAG vector [38] using Lipofectamine (Invitrogen). Then, they were washed and incubated with the transfection mixture for 6 h. Fresh media was then added and dynamics of Gag molecules was investigated 24 h later at 37 °C as described above.

### Ethics approval and consent to participate

Not applicable.

### Consent for publication

Not applicable.

### Availability of data and material

The datasets supporting the conclusions of this article are included within the article and its supplementary files.

## Additional files

Additional file 1: Figure S1. - Detection probability as a function of the segment length of confined or directed diffusion modes within a Brownian trajectory using BPNN. 200 trajectories of 450 frames including four segments with different lengths (20, 30, 40 and 50 frames) of a single mode of diffusion were analyzed (confined in light grey, directed in dark grey). The velocity is 1.5 μm/s for directed trajectories and the confinement diameter is 1 μm for confined trajectory (D = 0.25 μm^2^/s, integration time = 100 ms). The percentage of decision based on BPNN corresponds to the number of positive decision for a specific motion mode detected for a given frame over 200 trajectories and normalized to 1 or-1 for confined or directed trajectories, respectively. A 30 nm localization noise was added to the trajectory. (PDF 303 kb) Additional file 2: Figure S2. - Comparison of the percentage of decision using the BPNN, Hidden Markov Modeling (HMM)-Bayes, Bayesian Information Criterion (BIC) or Support Vector Machines (SVM) algorithms. 200 simulated trajectories of 300 frames mimicking diffusion within plasma membranes, including one directed motion segment with velocity randomly ranging from 1 to 3 μm/s and one confinement segment with diameters ranging from 0.5 and 1.2 μm, were analyzed with BPPN, HMM-Bayes, BIC or SVM. Within a trajectory each 50 frames segment is always localized at the same position. The diffusion coefficient D is 0.25 μm^2^/s and the integration time 100 ms. A 30 nm localization noise *Pn* was added to the trajectory (see Material and Methods section). The percentage of decision based on BPNN corresponds to the number of positive decision for a specific motion mode detected for a given frame over 200 trajectories and normalized to 1 or-1 for confined (light grey) or directed (dark grey) trajectories, respectively. The HMM-Bayes and the BIC algorithms can only detect directed or confined segments within a trajectory, respectively. The tables at the bottom detail the performance of the 4 algorithms in terms of sensitivity and specificity for detecting confined and directed motion modes in the range of parameters tested in this study (D = 0.25 μm^2^/s, 1 μm/s < v < 3 μm/s, 0.5 μm < L < 1.2 μm). (PDF 400 kb)
