# Supplementary figures and images for: Automatic detection of diffusion modes within biological membranes using back-propagation neural network
Source: BMC Bioinformatics. 2016 May 4;17:197. doi: 10.1186/s12859-016-1064-z (PMC4855490; doi:10.1186/s12859-016-1064-z)

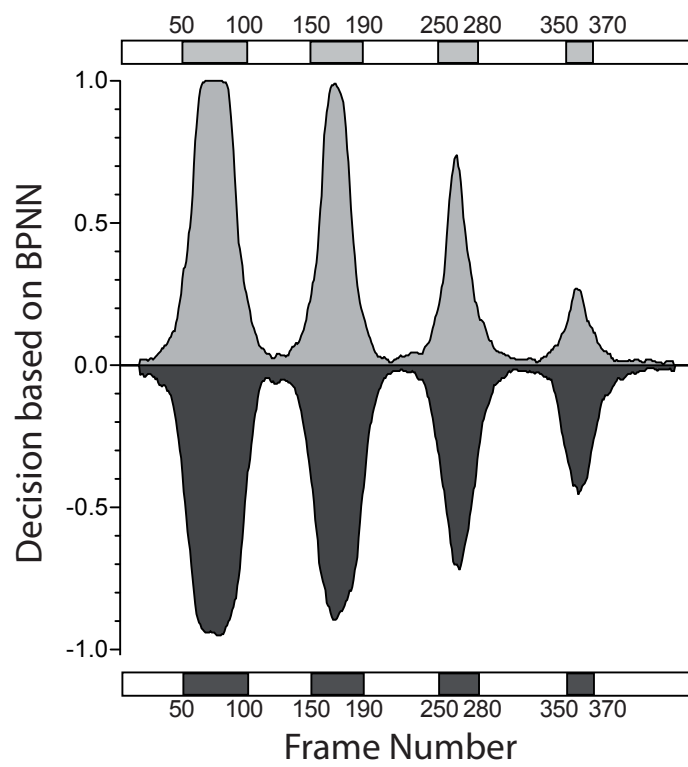

Supplement: Additional file 1: Figure S1. — - Detection probability as a function of the segment length of confined or directed diffusion modes within a Brownian trajectory using BPNN. 200 trajectories of 450 frames including four segments with different lengths (20, 30, 40 and 50 frames) of a single mode of diffusion were analyzed (confined in light grey, directed in dark grey). The velocity is 1.5 μm/s for directed trajectories and the confinement diameter is 1 μm for confined trajectory (D = 0.25 μm2/s, integration time = 100 ms). The percentage of decision based on BPNN corresponds to the number of positive decision for a specific motion mode detected for a given frame over 200 trajectories and normalized to 1 or-1 for confined or directed trajectories, respectively. A 30 nm localization noise was added to the trajectory. (PDF 303 kb) [file 12859_2016_1064_MOESM1_ESM.pdf]
