# Supplementary material for: Automatic detection of diffusion modes within biological membranes using back-propagation neural network
Source: BMC Bioinformatics. 2016 May 4;17:197. doi: 10.1186/s12859-016-1064-z (PMC4855490; doi:10.1186/s12859-016-1064-z)
Supplement: Additional file 2: Figure S2. — - Comparison of the percentage of decision using the BPNN, Hidden Markov Modeling (HMM)-Bayes, Bayesian Information Criterion (BIC) or Support Vector Machines (SVM) algorithms. 200 simulated trajectories of 300 frames mimicking diffusion within plasma membranes, including one directed motion segment with velocity randomly ranging from 1 to 3 μm/s and one confinement segment with diameters ranging from 0.5 and 1.2 μm, were analyzed with BPPN, HMM-Bayes, BIC or SVM. Within a trajectory each 50 frames segment is always localized at the same position. The diffusion coefficient D is 0.25 μm2/s and the integration time 100 ms. A 30 nm localization noise Pn was added to the trajectory (see Material and Methods section). The percentage of decision based on BPNN corresponds to the number of positive decision for a specific motion mode detected for a given frame over 200 trajectories and normalized to 1 or-1 for confined (light grey) or directed (dark grey) trajectories, respectively. The HMM-Bayes and the BIC algorithms can only detect directed or confined segments within a trajectory, respectively. The tables at the bottom detail the performance of the 4 algorithms in terms of sensitivity and specificity for detecting confined and directed motion modes in the range of parameters tested in this study (D = 0.25 μm2/s, 1 μm/s < v < 3 μm/s, 0.5 μm < L < 1.2 μm). (PDF 400 kb) [file 12859_2016_1064_MOESM2_ESM.pdf]

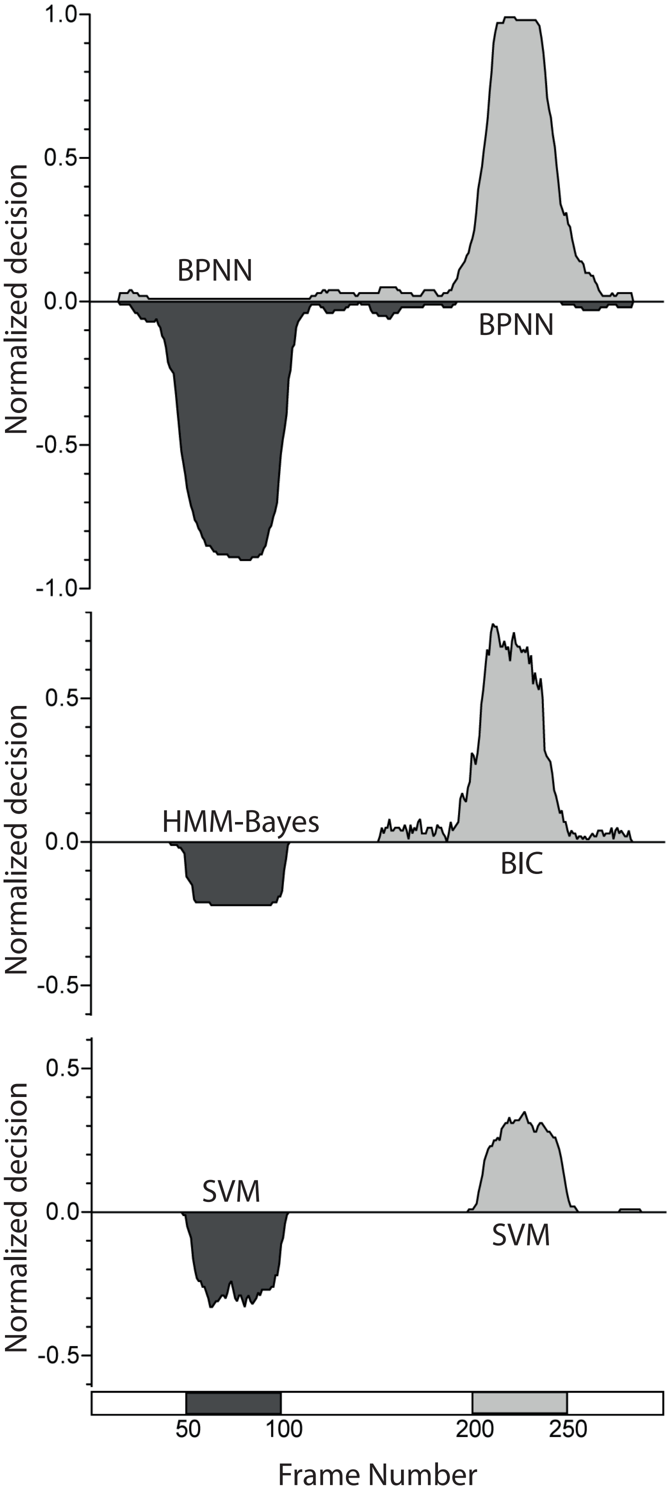

Directed motion	PaTrack	SVM	HMM-Bayes
Sensitivity (%)	83.1	26.1	20.9
Specificity (%)	95.4	99.9	99.5

Confined motion	PaTrack	SVM	BIC
Sensitivity (%)	75.3	24.5	49.9
Specificity (%)	96.8	99.9	95,0
